# Usability and Workflow Integration of a Machine Learning–Derived Neonatal Risk Predictor in Kenyan Neonatal Units: Multisite User-Centered Pilot Evaluation

**DOI:** 10.2196/94828

**Published:** 2026-09-09

**Authors:** Ronald Danny Nyatuka, Paul Macharia, Esther Kakhata, Faith Siva, Betsy Muriithi, Md Shafiqur Rahman Jabin

**Affiliations:** 1 School of Computing and Engineering Sciences Strathmore University Nairobi, Nairobi County Kenya; 2 Department of Medicine and Optometry Linnaeus University Kalmar, Kalmar Sweden; 3 Faculty of Health and Social Care University of Bradford Bradford, England United Kingdom

**Keywords:** health technology adoption, frontline health care workers, decision-support systems, early-life morbidity, cognitive load theory, documentation burden, health care quality improvement, mixed-methods evaluation, health systems strengthening, low-resource settings

## Abstract

**Background:**

Neonatal mortality remains a leading contributor to under-5 deaths globally, particularly in low- and middle-income countries (LMICs). While machine learning (ML)–based risk prediction models show promise for identifying high-risk neonates, published evidence describing the real-world usability and practical implementation of ML-derived neonatal risk prediction tools within routine clinical workflows in LMIC neonatal units remains limited.

**Objective:**

This study aimed to evaluate the usability, user experience, and perceived clinical utility of a paper-based neonatal risk predictor tool derived from an ML model and implemented across 3 Kenyan health facilities.

**Methods:**

A postimplementation, cross-sectional usability evaluation was conducted following a 4-month implementation period from August through November 2025. The study was embedded within a longitudinal mixed methods project. Frontline neonatal health care workers (n=10) completed standardized usability instruments, including adapted global usability items (System Usability Scale [SUS]), selected Questionnaire for User Interaction Satisfaction (QUIS) domains, and the Post-Study System Usability Questionnaire (PSSUQ), alongside a project-specific Post-Study Neonatal Utility Questionnaire (PSNUQ). Descriptive statistics (medians, IQRs, and category percentages) were computed. A total of 3 purposively selected neonatal unit leaders participated in semistructured key informant interviews (KIIs), which were analyzed using thematic analysis. Quantitative and qualitative findings were triangulated to contextualize perceptions of usability.

**Results:**

Among participating frontline health care workers, overall perceptions of usability were generally favorable. Around 75% (6/8) of respondents reported being willing to use the tool frequently, and 55% (5/9) indicated confidence in using it independently. Around half (5/10) disagreed that the tool was complex, while 22% (2/9) agreed, indicating moderate polarization in perceived complexity. Median PSSUQ composite scores were below 3 across subscales, reflecting positive usability ratings. Overall, 8 of 10 (80%) respondents agreed that the tool supports early identification of high-risk neonates and improves care prioritization within the first 48 hours. However, workflow integration was workload-sensitive: 40% (4/10) reported an increased documentation burden during periods of high patient volume. KIIs identified staffing shortages, parallel documentation systems, and the importance of administrative endorsement as key structural influences on adoption.

**Conclusions:**

The neonatal risk predictor tool demonstrated acceptable usability, learnability, and perceived clinical relevance across 3 diverse Kenyan facilities. However, variability in perceived complexity and workload sensitivity highlights the importance of structured onboarding, workflow-aligned integration, and context-aware implementation planning. These findings underscore that translating ML-derived predictor models into clinical practice requires not only technical validity but also strong usability and system-level readiness within routine neonatal care settings.

**International Registered Report Identifier (IRRID):**

RR2-10.2196/81996

## Introduction

Neonatal mortality remains a major global health challenge, accounting for almost half of all under-5 deaths worldwide, with an estimated 2.3 million newborn deaths in 2022 [1]. Despite global progress in reducing child mortality, declines in neonatal deaths have been slower and progress uneven across regions, making early identification and management of high-risk newborns a priority for achieving Sustainable Development Goal (SDG) Target 3.2 [2,3].

Structured, timely risk identification at birth and during the first 48 hours is critical because many life-threatening conditions (such as prematurity-related complications, intrapartum injury, and early-onset infection) present within this window [3]. In low- and middle-income country (LMIC) settings, reliance on subjective clinical judgment and inconsistent documentation often reduces the reliability of early triage, contributing to delays in escalation of care [3]. These realities create a pressing need for practicable risk-stratification tools that fit routine neonatal workflows and improve early detection of clinical deterioration.

Machine learning (ML) and other data-driven approaches show promise in neonatal risk prediction, with systematic reviews demonstrating that ML models can predict neonatal mortality and other adverse perinatal outcomes using routinely collected predictors [4-6]. However, most published ML models remain limited by modest external validation, variable predictor availability across settings, and sparse evidence on real-world deployment [4,5]. This gap between technical model development and safe operational deployment has also been documented in broader health information technology (HIT) literature, where system functionality issues, configuration errors, and workflow misalignment have resulted in unintended care disruptions [7-10]. Consequently, translating ML predictors into clinical practice requires 2 parallel lines of evidence: (1) demonstration of acceptable technical performance, and (2) evidence that the predictor tool is usable, acceptable, and practically feasible for frontline health care workers within the constraints of their working environments [6,11-13].

Usability and user experience (UX) are central determinants of health-technology adoption: internationally recognized standards define usability in terms of effectiveness, efficiency, and satisfaction in a specific context of use [9,14], and empirical work shows that poor usability undermines both uptake and the intended effect of clinical decision-support tools [14,15]. Evidence from national incident-report analyses indicates that poorly integrated digital systems often cause workflow disruptions, documentation inconsistencies, and patient safety risks when usability and contextual fit are not adequately addressed [8-10,16]. In health care, and especially in high-stress, time-sensitive clinical areas such as neonatal units, even small frictions in interface clarity, learnability, or workflow fit can materially reduce consistent use and thereby erode potential clinical benefit [9,14,15].

User-centered design (HCD) approaches that iteratively involve end-users in design, testing, and refinement increase the probability of adoption and sustained use of digital health tools in LMICs [17,18]. Prior work examining digital health testbeds and system configuration failures underscores the importance of early-stage contextual testing and iterative refinement to prevent downstream operational disruptions [9,10]. HCD emphasizes aligning an intervention’s information architecture, terminology, and workflow demands with the cognitive load and priorities of frontline staff, an especially important requirement when staffing shortages and high nurse-to-patient ratios constrain the time available for documentation [17]. For neonatal risk predictors, HCD therefore implies not only designing an accurate predictor tool but also ensuring the tool’s presentation, learning curve, and integration into routine tasks are acceptable to users [18-20].

Despite the theoretical consensus, there remains remarkably little real-world evidence on the usability and perceived utility of neonatal risk predictor tools in LMIC neonatal units [11]. Existing studies of ML-based neonatal prediction largely focus on model building and internal validation, with few studies examining frontline user perceptions, interface quality, and the practical trade-offs staff make when integrating new documentation tasks into busy clinical workflows [3,5]. This gap is consequential: without evidence that frontline users can and will use a predictor tool reliably, implementation at scale risks failure even if the underlying model performs well on retrospective data [3,5].

This study presents the completed user-centered evaluation of a neonatal risk predictor tool derived from an ML model [4] and implemented as a paper-based risk tool across 3 Kenyan health facilities, following the methodology outlined in the previously published study protocol [21]. Our objectives were to (1) assess perceived usability and user experience using validated instruments and bespoke UX items, (2) describe perceived usefulness and likely impact on routine neonatal workflows, and (3) identify design and training priorities to strengthen adoption in resource-constrained neonatal units. By focusing on frontline user perspectives and practical workflow constraints, the paper aims to provide the implementation-oriented evidence needed to move from ML model development toward safe, usable, and context-appropriate deployment.

## Methods

### Overview

The study adhered to good practices for pragmatic usability assessment in clinical settings. The study is reported in accordance with the Good Reporting of A Mixed Methods Study (GRAMMS) recommendations [22], and the completed GRAMMS checklist is provided as Multimedia Appendix 1.

### Study Design

This study used a postimplementation, cross-sectional usability, and UX evaluation embedded within a longitudinal mixed-methods project. The use of mixed methods triangulation aligns with established approaches for evaluating complex health care interventions, combining quantitative usability metrics with qualitative contextual insight to enhance interpretive depth [23-25]. The evaluation combined standardized usability instruments with a project-specific Post-Study Neonatal Utility Questionnaire (PSNUQ) and semistructured key informant interviews (KIIs) to triangulate frontline users’ perceptions with contextual implementation data. The quantitative usability component reported here was administered after a 4-month implementation period of the paper-based neonatal risk predictor tool. The study followed good practice for pragmatic usability assessment in clinical settings [14].

**Figure 1:**
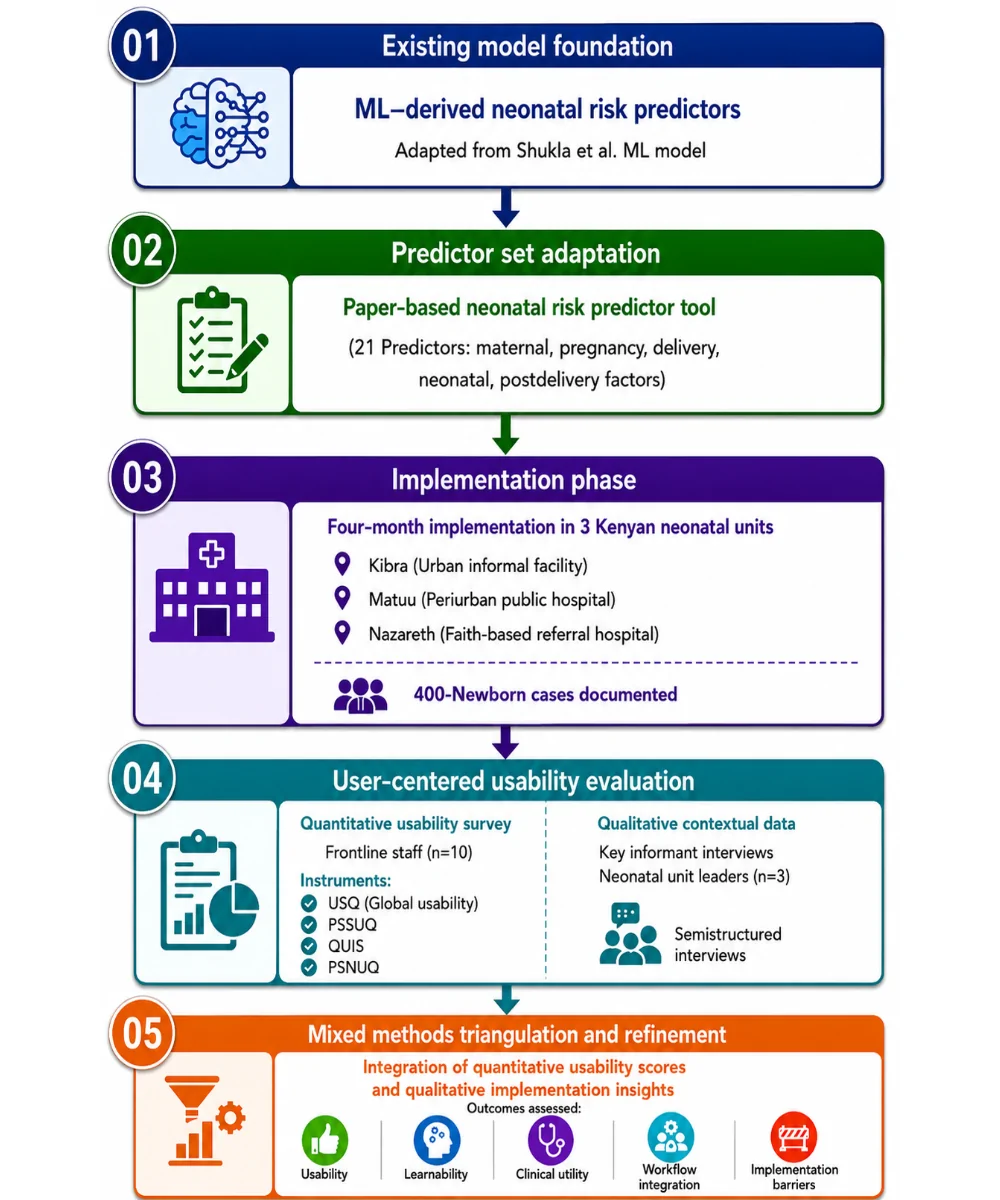
Study design and mixed methods usability evaluation framework. ML: machine learning; PSNUQ: Post-Study Neonatal Utility Questionnaire; PSSUQ: Post-Study System Usability Questionnaire; QUIS: Questionnaire for User Interaction Satisfaction; USQ: Usability Scale Questionnaire.

This study represents the completed user-centered usability evaluation described in the previously published study protocol [21]. This paper reports the implementation and postimplementation usability findings from that protocol-defined study. Preintervention key informant interviews were conducted in August 2025, followed by implementation of the paper-based neonatal risk predictor and newborn data collection from August through November 2025. The postimplementation usability survey was administered in December 2025. The overall study design and mixed-methods usability evaluation framework are illustrated in Figure 1.

The ML–derived neonatal risk predictor tool was implemented as a paper-based documentation instrument across 3 Kenyan neonatal units over a 4-month period. Following implementation, usability and user experience were evaluated using a mixed methods approach that combined standardized usability questionnaires (System Usability Scale [SUS], Post-Study System Usability Questionnaire [PSSUQ], Questionnaire for User Interaction Satisfaction [QUIS], and PSNUQ) completed by frontline neonatal staff (n=10) with qualitative KIIs with neonatal unit leaders (n=3). Quantitative and qualitative findings were triangulated to assess usability, learnability, clinical utility, workflow integration, and implementation context.

### Study Setting

The study was conducted in 3 Kenyan health facilities representing distinct service contexts: an urban informal settlement facility in Kibra (Nairobi County), a periurban public hospital in Matuu (Machakos County), and a faith-based referral hospital in Nazareth (Kiambu County). Facilities were located in Nairobi County (Kibra), Machakos County (Matuu), and Kiambu County (Nazareth). Contextual details and facility characteristics are described in the project report and site summaries.

### Participants and Sampling

The published study protocol specified a purposive sample of 10 health care workers across the 3 participating facilities, with a planned allocation of 4 participants from Kibera, 4 from Matuu, and 2 from Nazareth [21]. In the completed study, however, recruitment was based on the availability of eligible frontline neonatal health care workers during the implementation period. All eligible and available staff involved in the implementation were invited to participate, and 10 frontline staff completed the postimplementation survey.

The protocol’s prespecified allocation of 4 participants from Kibra, 4 from Matuu, and 2 from Nazareth was intended to ensure representation across the 3 participating facilities. The allocation was a planned recruitment target rather than a statistically derived proportional allocation. In the completed study, the final numbers of participants from each facility corresponded to these planned numbers, although recruitment was based on the availability of eligible frontline neonatal health care workers during the implementation period.

A total of 2 participant groups contributed to the usability evaluation:

1. Key informants (qualitative KIIs): Purposive sampling was used to select senior neonatal unit leaders (one per facility) for semistructured interviews exploring implementation context, governance structures, workflow integration, and perceived barriers and enablers to adoption. A total of 3 KIIs were conducted across the participating facilities. These interviews have also been reported in a separate published analysis examining governance and health system determinants of scalability and institutionalization of the neonatal risk stratification tool [26]. In this study, the same interview data were used to contextualize the user-centered evaluation, with analysis focused specifically on perceptions of usability, workflow integration, and implementation-related factors relevant to the frontline use of the paper-based predictor tool. The semistructured interview guide is provided in
  Multimedia Appendix 2
  .
2. Frontline neonatal health care workers (quantitative postsurvey): The 3 participating facilities had a combined workforce of 43 neonatal health care workers: 11 at Kibra, 15 at Matuu, and 17 at Nazareth. All eligible neonatal nurses and midwives involved in the 4-month implementation of the tool were invited to complete the post-implementation survey. Due to staff availability during the study period, 10 frontline health care workers participated in the survey. Participants were distributed across the 3 facilities as follows: 4 from Matuu, 4 from Kibra, and 2 from Nazareth.

All participants provided information and consent for the use of anonymized aggregate responses in research outputs.

### Development of the ML-Derived Neonatal Risk Predictor

The paper-based neonatal risk predictor evaluated in this study was derived from a previously developed and validated ML model for neonatal mortality prediction reported by Shukla et al [4]. The original model was developed using routinely collected maternal, obstetric, delivery, neonatal, and early postnatal variables and demonstrated good predictive performance in LMIC settings (area under the receiver operating characteristic curve >0.80). Rather than evaluating the predictive performance of the ML algorithm itself, this study focused on the feasibility, usability, user experience, and workflow integration of a paper-based implementation of the predictor set within routine neonatal care across 3 Kenyan health facilities.

The complete list of predictor variables and the paper-based neonatal risk predictor form used during implementation are provided in Multimedia Appendix 2.

### Intervention and Exposure

The object of evaluation was a paper-based neonatal risk predictor tool (the “predictors set”) that had been integrated into routine documentation for newborns at delivery (Day 1) and at a postdelivery check (Day 2). The tool captured 21 candidate predictors, organized into 5 domains: maternal characteristics, pregnancy-related factors, delivery characteristics, neonatal clinical indicators, and early postdelivery observations. The complete list of predictors and the paper-based neonatal risk predictor form are provided in Multimedia Appendix 2 to enhance transparency and reproducibility. During the 4-month implementation period, the tool was used prospectively within routine neonatal care, resulting in 400 newborn cases recorded across the 3 participating sites: 100 at Kibra, 100 at Nazareth, and 200 at Matuu. These site-specific totals represent the cases captured during the implementation period and were not determined through a statistically derived per-site sampling allocation.

### Instruments

#### Standard Instruments

We combined well-established usability measures to capture complementary aspects of usability and user experience:

1. PSSUQ: The PSSUQ (James R Lewis) is a validated instrument designed to measure users’ perceived system usability after task completion; the most commonly used short form has 16 items across dimensions such as system usefulness, information quality, and interface quality, typically scored on a 7-point Likert scale (lower scores = better perceived usability). The PSSUQ has been psychometrically evaluated and widely used in health technology research [15,27]. PSSUQ scores were summarized using the standard System Usefulness, Information Quality, and Interface Quality subscales, together with the overall score.
2. QUIS: The QUIS is a user satisfaction instrument developed by Chin et al [28] that measures overall reaction and multiple interface dimensions (eg, screen factors, terminology, and learnability). We used QUIS items to assess interaction satisfaction and perceptions of content and layout [28]. Selected QUIS domains assessed overall user satisfaction, ease of use, interface characteristics, and learning.
3. SUS: While our project labels one instrument “SUS” in the field documents, the SUS is the canonical short usability scale commonly used to obtain a global usability score (10 items and 5-point Likert) [29]. In our implementation, we used a short version of SUS, adapted from standard global usability measures (SUS and PSSUQ constructs), to capture overall perceived ease of use and willingness to reuse.

#### Project-Specific Instrument Content (PSNUQ and SUS Items)

The PSNUQ includes domains on perceived usefulness, learnability, task support, content quality, interface quality, and perceived productivity and impact. The SUS items in the survey covered willingness to use, perceived complexity, need for assistance, and overall ease of use. The QUIS subscales assessed overall reaction, layout and text clarity, terminology, and interaction satisfaction.

#### Questionnaire Administration and Data Collection Procedures

Postsurvey questionnaires were administered face-to-face by the research team to frontline neonatal staff at the end of the implementation period. Respondents completed the combined instrument (SUS+QUIS+PSNUQ) in a private setting; the survey took approximately 20-30 minutes. KIIs with neonatal unit leads were conducted using a semistructured guide, with audio recording with permission; interview transcripts were used for thematic analysis and triangulation.

The complete questionnaires, including all item wording, response anchors, scoring procedures, and the PSSUQ subscale allocation, are provided in Multimedia Appendix 2. The SUS, PSSUQ, and selected QUIS domains are validated usability instruments, whereas the PSNUQ was developed for this study to evaluate perceived clinical relevance, feasibility, and implementation considerations of the neonatal risk predictor tool.

#### Scoring and Quantitative Analysis

Survey items used Likert-style ordinal response scales (typically 5- or 7-point, depending on the instrument and item). For standardized instruments (PSSUQ, QUIS, and SUS-derived items), we followed canonical scoring procedures where applicable (eg, SUS scoring and PSSUQ subscales) and computed descriptive statistics (median, IQR, and category percentages) for each item and subscale. Given the small sample size (n=10), analyses were descriptive, focusing on response distributions, identifying polarized items, and cross-tabulations by facility or cadre where numbers allowed. We also computed simple case-level completeness and item-level missingness for the survey dataset. These methods align with pragmatic usability evaluation approaches in clinical contexts [14,15].

#### Qualitative Analysis and Triangulation

KIIs were audio-recorded with participant consent and transcribed verbatim. The transcripts were analyzed using thematic analysis through an iterative coding process, with coding conducted manually using a structured coding framework informed by the interview guide and patterns emerging from the data [30,31]. EK and FS conducted the initial coding, while RDN and MSRJ independently reviewed the coding and emerging themes. Coding discrepancies were discussed among the coders and resolved through consensus. The analysis focused on identifying themes relevant to usability, workflow integration, and implementation context, with the qualitative findings used primarily to contextualize and triangulate the quantitative usability findings rather than as a standalone qualitative outcome analysis. In particular, qualitative insights helped explain divergent or polarized survey responses, including concerns related to staffing shortages, documentation duplication, workload pressure, infrastructure limitations, and resistance to workflow changes. The researchers acknowledged that interpretation of the qualitative data was informed by their experience in digital health and neonatal care, and efforts were made to minimize interpretive bias through regular discussion of emerging themes. Given the exploratory nature of this pilot study and the inclusion of one key informant from each participating facility, thematic saturation was not formally assessed. Representative deidentified quotations supporting the themes are provided in Multimedia Appendix 3. Findings from the quantitative postsurvey (SUS, QUIS, and PSNUQ components) and the qualitative KIIs were then triangulated to generate integrated interpretations. This triangulation enabled identification of both tool-specific usability issues (eg, clarity, learnability, and layout) and system-level constraints affecting adoption (eg, staffing capacity, governance processes, and infrastructure readiness). The triangulated findings informed practical recommendations for design refinement and implementation planning.

The reporting checklist, complete postimplementation survey instrument (SUS, selected QUIS domains, PSSUQ items, and PSNUQ), semistructured interview guide, and the paper-based neonatal risk predictor form are provided as Multimedia Appendices 1-3 to support transparency and reproducibility.

### Ethical Considerations

Ethical approval (SU-ISERC2949/25) was obtained from the Strathmore University Institutional Scientific Ethics Review Committee (SU-ISERC), Nairobi, Kenya, on 28 July 2025, prior to commencement of the study. All participants provided informed consent before participation after receiving information about the study aims, voluntary participation, confidentiality protections, and data management procedures. Interview transcripts were anonymized before analysis to protect participant confidentiality and institutional privacy. The study adhered to established ethical principles for qualitative health systems research throughout data collection, analysis, and reporting.

## Results

### Participant Characteristics

A total of 10 frontline neonatal health care workers completed the postintervention usability evaluation across the 3 study sites. The majority of respondents were mid-career nurses with diploma-level qualifications, reflecting the cadre primarily responsible for routine neonatal documentation and triage. The characteristics and study-site distribution of the participating frontline neonatal health care workers are summarized in Table 1.

**Table 1:** Characteristics of frontline neonatal health care worker participants (n=10).

Characteristics		Values, n (%)^a^
Study site (n=10)		
	Kibra	4 (40)
	Matuu	4 (40)
	Nazareth	2 (20)
Professional cadre (n=10)		
	Nurses	8 (80)
	Midwives	2 (20)
Age (years; n=9)		
	18-30	2 (22.2)
	31-40	6 (66.7)
	51-60	1 (11.1)
Educational qualification (n=9)		
	Certificate	7 (77.8)
	Bachelor's degree	2 (22.2)

^a^Percentages are calculated using the number of respondents for each characteristic. One participant had missing responses for age and educational qualification; therefore, the denominator for these variables is 9 rather than 10.

### Overall Usability Perception: Global Usability (SUS Items)

Among participating frontline health care workers, overall perceptions of usability were generally favorable. Most respondents reported positive overall perceptions of the neonatal risk predictor tool's usability. Specifically, 75% (6/8) reported a willingness to use the tool frequently in routine practice, and 55% (5/9) indicated confidence in using it independently. Regarding perceived complexity, around half (5/10) disagreed that the tool was complex, while 22% (2/9) agreed. Together, these findings suggest the tool's overall acceptability, with moderate variability in users' perceptions of complexity.

### PSSUQ Composite Scores

PSSUQ subscales were computed using canonical scoring procedures (lower scores indicate better usability). Median scores across all subscales were below 3, indicating favorable perceptions of usability.

Information quality and interface quality showed slightly greater dispersion, reflecting variability in respondents’ perceptions of clarity and layout. Median PSSUQ subscale scores are presented in Table 2. All median scores were below 3, indicating generally favorable perceptions of system usability.

**Table 2:** PSSUQ subscale scores.

Subscale	Values, median (IQR)^a^
System Usefulness	2.1 (1.5-2.8)
Information Quality	2.3 (1.7-3.0)
Interface Quality	2.4 (1.8-3.2)
Overall PSSUQ^b^ score	2.3 (1.6-3.0)

^a^PSSUQ responses were available for 10 participants (n=10). Scores are presented as median (IQR). Lower scores indicate greater perceived usability.

^b^PSSUQ: Post-Study System Usability Questionnaire.

### User Experience Domains (QUIS and PSNUQ)

#### Content and Layout Clarity

A majority of respondents (8/10, 80%) agreed that the content of the tool was clearly structured, and the same proportion (8/10, 80%) indicated that the terminology was understandable. Additionally, 7 respondents (7/10, 70%) agreed that the information was logically grouped. However, 3 respondents (3/10, 30%) expressed concerns regarding potential redundancy with existing documentation forms.

#### Learnability and Confidence

A total of 8 respondents (8/10, 80%) agreed that the neonatal risk predictor tool was easy to learn after initial exposure, whereas 2 respondents (2/10, 20%) reported some initial difficulty during early use. Regarding confidence in using the tool independently, 6 respondents (6/10, 60%) indicated that they felt confident completing the tool without assistance following the implementation period, while 4 respondents (4/10, 40%) reported requiring occasional clarification or support during early stages of use.

In addition, 7 respondents (7/10, 70%) reported that their comfort level with the tool increased over the 4-month implementation period, suggesting progressive familiarization with the structured documentation format. These findings indicate that while initial onboarding may require guidance for a subset of users, perceived learnability improved with continued exposure.

#### Perceived Clinical Utility

PSNUQ responses demonstrated high perceived clinical utility of the neonatal risk predictor tool. A total of 8 respondents (8/10, 80%) agreed that the tool supports early identification of high-risk neonates, while 7 respondents (7/10, 70%) agreed that it enhances structured clinical thinking during neonatal assessment. Additionally, 8 respondents (8/10, 80%) reported that the tool may improve care prioritization within the first 48 hours of life.

#### Workflow Integration

Although the tool was perceived as clinically helpful, integration into the routine workflow was described as dependent on staffing capacity. Overall, 6 respondents (6/10, 60%) indicated that the tool was manageable under normal staffing conditions, whereas 4 respondents (4/10, 40%) reported that high patient volumes increased the perceived documentation burden. Among those reporting higher-workload environments, 3 respondents (3/10, 30%) specifically noted that competing clinical priorities made consistent completion more challenging.

#### Polarization and Divergent Perceptions

Item-level distribution analysis demonstrated moderate polarization on complexity-related items. Item-level responses demonstrated moderate variation in perceptions of tool complexity. A total of 5 respondents (5/10, 50%) disagreed or strongly disagreed that the tool was complex, 3 (3/10, 30%) selected a neutral response, and 2 (2/10, 20%) agreed or strongly agreed.

### Triangulated Interpretation

Qualitative insights from KIIs provided contextual interpretation of the usability findings. All 3 informants (n=3, 100%) identified staffing shortages as a structural factor influencing perceived documentation burden. A total of 2 informants (n=3, 67%) reported that parallel documentation requirements contributed to concerns about duplication, particularly in facilities where existing paper records must be maintained alongside new tools. Additionally, 2 informants (n=3, 67%) emphasized that visible administrative endorsement influenced staff motivation to adopt and consistently use the tool.

Triangulation of quantitative survey findings with qualitative themes indicates that perceived usability was shaped not only by interface clarity and learnability but also by broader structural workflow conditions within the neonatal units.

In Summary, the neonatal risk predictor tool demonstrated favorable usability performance across multiple domains. Survey responses indicated generally positive global usability ratings, acceptable learnability following initial exposure, and strong perceived clinical relevance for the early identification and prioritization of high-risk neonates. At the same time, item-level distribution revealed moderate variability in perceptions of complexity, with a subset of respondents reporting increased burden under high-workload conditions. These patterns collectively reflect broad usability across participants, alongside measurable differences in experience related to workflow intensity and staffing context.

## Discussion

### Principal Findings and Interpretation

This pilot, multisite usability evaluation suggests that the neonatal risk predictor tool was generally perceived as usable, learnable, and clinically relevant by participating frontline neonatal health care workers across the 3 Kenyan facilities. Most respondents reported willingness to use the tool regularly and increasing confidence with continued exposure; median composite usability scores were in the favorable range. At the same time, item-level responses revealed measurable polarization on perceived complexity and workload sensitivity, with a minority of users reporting initial difficulty and persistent concerns about documentation burden under high patient loads. These findings suggest that while the tool is broadly acceptable to users, its successful integration into routine neonatal workflows will depend on workload-aware implementation strategies and structured onboarding.

Our results align with broader evidence emphasizing the importance of usability and contextual fit for clinical decision-support tools in LMICs. The neonatal period remains the most vulnerable window for child survival globally, and interventions that improve early identification of high-risk neonates must be operationally feasible at the point of care to affect outcomes [1,3]. The finding that frontline health care workers value clinically relevant predictors (eg, Appearance, Pulse, Grimace, Activity, and Respiration [APGAR], gestational age, and vital signs) is consistent with prior ML prediction reviews that identify these variables as strong, widely used predictors of neonatal outcomes [5]. However, systematic reviews and expert commentaries have repeatedly highlighted that technical model performance alone is insufficient for impact: external validation, predictor availability, and deployment logistics are essential for translation [5]. Our triangulated usability and KII data confirm this; technical validity must be matched by usability and health-system readiness for effective adoption [3,5].

Our findings are also consistent with previous usability evaluations of digital health interventions in maternal and neonatal care. For example, Balderas-Díaz et al [32] demonstrated that user-centered design, iterative proof-of-concept evaluation, and usability testing are essential for promoting user acceptance and successful implementation of adaptive mHealth systems in perinatal care. Consistent with their findings, our study highlights that, beyond technical performance, successful adoption depends on usability, workflow integration, and alignment with users' clinical needs.

### Usability Implications and Standards

The observed variability in perceived complexity and information-quality dispersion highlights the need to follow established usability standards and HCD principles when refining and scaling the tool. ISO 9241-11 defines usability as effectiveness, efficiency, and satisfaction in a specified context of use; our findings show acceptable satisfaction and improved efficiency with use, but situational constraints reduce effectiveness in high-workload settings [14]. Similarly, reviews of usability instruments in mHealth underline the importance of combining standardized psychometric measures (eg, PSSUQ, QUIS, and SUS) with context-specific items that capture workflow and documentation interactions [15]. Our approach, combining standardized scales with a project-specific PSNUQ and KIIs, therefore, aligns with recommended practice for pragmatic usability evaluation in health settings [14,15].

### Interpretation of Variability and Polarization

The observed polarization in perceived complexity is not merely noise—it is a signal that the intervention interacts with heterogeneous user contexts, capacities, and expectations. Some frontline staff found the tool intuitive and readily incorporated it into routine care, while others experienced friction that increased perceived burden. This pattern commonly appears in early-stage usability evaluations and often reflects differences in prior exposure to structured documentation, individual triage strategies, and local workflow realities (eg, staffing and shift patterns). Interpreting polarization, therefore, requires moving beyond averages to examine subgroups (by facility, cadre, or prior digital familiarity) and to identify threshold conditions: small increases in caseload or complexity may flip a user from “comfortable” to “overloaded,” producing bimodal response distributions. Empirically, such subgroup heterogeneity is a cue for targeted refinements (eg, simplified pathways for high-workload contexts) rather than wholesale redesign [33].

From a theoretical perspective, polarization can also reflect differences in cognitive load and schema availability: users with established mental models for structured documentation experience lower intrinsic and extraneous cognitive load and therefore report lower perceived complexity; those lacking such schemas are more affected by extraneous load and report higher complexity (cognitive load theory). Targeted training that promotes rapid schema formation (eg, worked examples and supervised practice) is therefore likely to reduce polarization by elevating baseline competence across the team [34].

Finally, polarization carries implementation implications: rather than treating the tool as binary (adopt vs reject), implementation plans should include adaptive pathways, for example, a “light” checklist for high-pressure shifts and a richer version for less busy times, to maintain fidelity while accounting for context. This approach preserves core clinical value while lowering the activation energy for use under strain (implementation outcomes and feasibility guidance) [35].

### Translation Gap Between ML and Clinical Workflow

The literature on ML for neonatal prediction is clear: many models demonstrate high retrospective performance but fail to translate into routine care due to gaps in predictor availability, data quality, and workflow fit (external validation, calibration, and deployment are often missing). In other words, algorithmic accuracy is necessary but not sufficient for clinical impact; the “last mile” is practical integration into health care workers’ daily tasks [9,10]. This study therefore complements previous model-development research by evaluating the implementation of an ML-derived predictor set under routine clinical conditions rather than reassessing algorithmic performance. Consistent with broader analyses of health information technology incidents, system-level fragilities often emerge not from algorithmic logic itself but from workflow integration failures, configuration issues, and documentation burdens.

Our findings reinforce this translation gap: frontline staff valued clinically relevant predictors (APGAR, gestational age [GA], and vitals), but even clinically sensible predictors must be routinely and rapidly available in usable form for an ML-based tool to function in practice. This reinforces calls in the literature for deployment-focused research that explicitly couples model development with operational capture and usability testing [5,36].

Operational requirements for ML deployment, therefore, extend beyond model metrics to include measures of data completeness, real-time availability, and process fit. Our mixed results, reasonable data capture for many predictors but workload-sensitive usability, illustrate why ML systems often falter post deployment: data pipelines may look good in retrospective datasets but break when frontline realities impose delays, missing fields, or inconsistent recording habits. Addressing this requires co-designed data workflows, automated data extraction when possible, and continuous monitoring of data quality during deployment [5,37].

Although the neonatal risk predictor evaluated in this study was derived from a validated ML model, participants were not asked to evaluate or compare the underlying algorithm. Instead, the usability evaluation focused on frontline users' experiences with the paper-based implementation of the predictor set within routine clinical workflows. Consequently, the findings should be interpreted as evidence regarding implementation and usability rather than perceptions of algorithmic transparency or trust in specific ML approaches.

### Cognitive and Workflow Load Considerations

Clinical tasks in neonatal units are time-pressured and cognitively intensive. Cognitive load theory highlights that working memory is limited and that extraneous load (poorly designed interfaces, unnecessary duplication) reduces the capacity available for core clinical reasoning. Our results, which showed that some users reported perceived complexity and workload-related friction, map precisely onto this theory. In practice, design choices that reduce extraneous cognitive load (prefilled defaults, binary checkboxes rather than free-text, and minimized decision branches) and scaffold intrinsic load (clear prompts and quick-reference thresholds) are most likely to improve consistent uptake. Empirical work on electronic health records (EHRs) and clinical decision support system **(**CDSS) shows that poor interface design increases cognitive load and error risk; conversely, simplified, context-aware interfaces reduce time-on-task and decision fatigue [34,38].

From a workflow standpoint, the tool must respect “attention budgets.” During high-volume shifts, health care workers have very limited time to document; even an additional 30 seconds per baby can be prohibitive. Therefore, interventions should be evaluated in terms of marginal time cost under realistic caseloads (time-motion studies), and design should aim to minimize per-case time through automation, task reallocation, or condensed item sets for critical screening. This attention to microeconomics of time turns usability into an outcome (time cost and adherence), not just a perception metric [38,39].

Our findings can also be interpreted through the Systems Engineering Initiative for Patient Safety (SEIPS) framework, which conceptualizes health care quality and patient safety as emerging from interactions among people, tasks, tools, and technologies, the physical environment, and organizational conditions [40,41]. From a SEIPS perspective, the documentation burden reported during periods of high workload reflects a work-system issue rather than a limitation of the neonatal risk predictor itself. Similarly, the qualitative findings regarding staffing shortages, parallel documentation systems, and organizational support indicate that successful implementation depends not only on the tool's usability but also on optimizing the broader clinical work system and integrating workflows. In low-resource settings, these considerations extend to the availability and reliability of the infrastructure supporting digital health implementation; evidence from Kenyan health facilities has highlighted the importance of addressing electricity and connectivity constraints and considering context-appropriate approaches to maintain continuity of digital health services [42,43].

### Implementation Science Perspective

Implementation science provides conceptual tools to interpret our findings and plan next steps. The Implementation Outcomes Framework (Proctor et al [35]) identifies acceptability, feasibility, appropriateness, and adoption as proximal outcomes that must precede penetration and sustainability. Our data show reasonable acceptability and perceived appropriateness, but feasibility and adoption are conditional on staffing and documentation realities, precisely the intermediate implementation outcomes that predict later penetration and sustainability. Framing results in this taxonomy clarifies operational priorities (eg, improving feasibility via workflow changes) and defines measurable implementation end points for subsequent trials.

Normalization process theory is also useful: it articulates how new practices become embedded through coherence (sense-making), cognitive participation (engagement), collective action (operational work), and reflexive monitoring [44]. Our KIIs and survey data indicate that coherence exists (staff see the value), but cognitive participation and collective action are hindered by workload and governance barriers, meaning that embedding will require active facilitation, local champions, and adaptive workflows to transform individual acceptance into routinized practice. These theoretical lenses suggest concrete implementation strategies (champion identification, cocreation, iterative feedback loops, and fidelity monitoring) that align with our empirical patterns [45].

### Contribution to LMIC Digital Health Literature

This study contributes, both practically and conceptually, to the emerging LMIC digital health literature in several ways. First, it presents real-world usability evidence from neonatal units, a high-impact but under-studied clinical domain in LMICs, and ties usability to operational capture metrics, bridging a common gap between model-focused and deployment-focused work [39]. Second, by triangulating standardized usability instruments with context-specific qualitative insights, the work demonstrates a pragmatic mixed methods evaluation blueprint that other LMIC implementers can replicate. Third, it underscores that pilot usability samples (n≈5-15) are methodologically defensible for early user-centered design validation while pointing toward necessary next steps (larger trials and psychometric validation), thereby setting realistic methodological expectations for the field. Finally, it emphasizes implementation equity: without attention to staffing, governance, and data burden, digital tools risk widening care quality gaps across facilities [46]. Digital inequities and differential adoption across populations have been documented in other contexts, where infrastructure, governance, and sociocultural factors shape digital uptake [47,48]. These contributions respond directly to calls in recent mHealth and implementation reviews for context-sensitive, deployment-oriented evidence.

### Implications for Practice

Based on our findings, implementers and facility managers should consider the following practical steps before scaling:

1. Structured onboarding and mentorship: Provide brief, practical training sessions with supervised use during the initial weeks and ongoing mentorship for new or rotating staff to reduce early-use errors and perceived complexity [49]. Evidence from health care quality improvement and digital training interventions demonstrates that structured onboarding and supervised adoption phases improve implementation fidelity and long-term usability outcomes [23,50,51].
2. Workflow-sensitive adaptation: Integrate the tool into existing delivery registers or consolidate parallel documentation forms to reduce duplication; where duplication is unavoidable for regulatory reasons, design the tool to minimize additional data entry (eg, checkboxes and prefilled fields) [52].
3. Task allocation and scheduling: Assign clear responsibilities (eg, primary recorder during delivery and secondary verifier) to ensure consistent completion during peak periods [53].
4. Hybrid implementation planning: For facilities with unreliable IT and infrastructure, maintain a hybrid approach (paper with planned digitization) and plan for offline digital options with synchronization capability [52,54].
5. Monitoring and iteration: Embed short feedback cycles (weekly and monthly) to capture user problems and quickly iterate on interface or process changes [54,55].
6. User engagement and co-design: Actively involve frontline health care workers in iterative refinement of the tool through feedback sessions, participatory design workshops, or pilot discussions. Engaging end users in the adaptation process can enhance local ownership, improve contextual fit, and increase the likelihood of sustained adoption within routine clinical workflows [17,20].

These pragmatic steps align with implementation science principles and can increase the likelihood that technically valid predictor models translate into practice improvements.

### Strengths and Limitations of the Study

Several features increase the study’s utility to researchers and implementers: (1) real-world exposure, that is, the usability assessment followed a 4-month implementation period rather than a short laboratory test, increasing ecological validity); (2) multisite design, that is, 3 diverse facility contexts (urban informal, periurban public, and faith-based referral) enhance transferability of implementation insights across LMIC delivery settings; (3) triangulated methods, that is, combining standardized usability instruments (SUS, QUIS and PSSUQ-derived items) with KIIs provided depth and helped explain quantitative polarization; and (4) operational realism, that is, the usability evaluation was explicitly tied to routine documentation and staffing realities, yielding actionable recommendations for onboarding and workflow adaptation.

We must acknowledge several limitations that constrain the generalizability and strength of conclusions. First, the postsurvey sample size was small (n=10), limiting statistical power and precluding robust inferential testing; this study should therefore be understood as a pilot usability evaluation rather than a definitive validation. Accordingly, the findings should be interpreted as preliminary evidence intended to inform future larger-scale usability and implementation studies rather than to support broad generalization. Second, although multi-site, qualitative key informant data were limited to 3 senior health care workers (n=3), which constrains claims about saturation for broad thematic generalization; however, those informants were purposively selected leaders with facility-level oversight, increasing the relevance of governance and workflow insights.

Third, the usability measures relied on self-report; objective measures of time on task, error rates, or direct observation could provide complementary evidence of efficiency and effectiveness. Fourth, the PSNUQ instrument is study-adapted; while combining it with validated scales strengthens construct coverage, formal psychometric validation (eg, reliability and factor structure) was not possible with this sample. Finally, the study used a paper-based implementation of the predictor tool; findings may differ with a fully digital interface and therefore should not be assumed to directly generalize to other delivery modalities. These limitations should guide the interpretation and design of future studies.

### Future Research Recommendations

To build on these pilot findings, we recommend:

1. Larger multisite usability and effectiveness trials: Expand sample sizes across more facilities and include objective time-motion measures, task completion rates, and downstream clinical process outcomes (eg, time to antibiotic administration, referral timeliness) [56].
2. Psychometric validation of PSNUQ: With a larger sample, assess internal consistency (Cronbach α) and factor structure to formally validate the adapted instrument [37].
3. Compare delivery modalities: Evaluate differences between paper, hybrid, and fully digital interfaces for the same predictor tool to identify modality-specific strengths and weaknesses [57].
4. Implementation trials linked to process outcomes: Conduct stepped-wedge or cluster-randomized evaluations to test whether increased usability and workflow integration translate into measurable improvements in neonatal care processes and, ultimately, clinical outcomes [58].
5. Cost-effectiveness and sustainability analysis: Assess the resource and financing implications of scaling, including staff time, training costs, and information communication technology (ICT) investments. These analyses will help policymakers weigh trade-offs between manual and digital approaches [59].

### Conclusions

This pilot usability evaluation suggests that participating frontline neonatal health care workers generally perceived the neonatal risk predictor tool as usable, learnable, and clinically relevant, while also identifying workflow-sensitive barriers, particularly staffing pressures and documentation duplication, that may influence implementation. These findings support proceeding to larger, methodologically rigorous evaluations while simultaneously investing in pragmatic onboarding, workflow integration, and infrastructure planning to maximize the potential for real-world impact. Given the global priority of reducing neonatal mortality, pragmatic usability evidence such as ours is a necessary step toward responsibly translating ML into safer, more timely neonatal care.

## Data Availability

The datasets generated and analyzed during the current study are not publicly available due to ethical and confidentiality restrictions. The data include deidentified qualitative interview transcripts collected from healthcare professionals within specific health facilities in Kenya. Given the small sample size and context-specific nature of the facilities, public sharing could potentially compromise participant confidentiality. Deidentified data may be made available from the corresponding author upon reasonable request, subject to institutional and ethical approval and applicable data protection requirements.

## Appendix

Multimedia Appendix 1 GRAMMS Checklist.

Multimedia Appendix 2 Data collection tools.

Multimedia Appendix 3 Qualitative verbatim quotes.

## Abbreviations

APGAR: Appearance, Pulse, Grimace, Activity, and Respiration
CDSS: clinical decision support system
EHR: electronic health record
GRAMMS: Good Reporting of A Mixed Methods Study
HCD: user-centered design
HIT: health information technology
ICT: information communication technology
KII: key informant interview
LMIC: low- and middle-income countries
ML: machine learning
PSNUQ: Post-Study Neonatal Utility Questionnaire
PSSUQ: Post-Study System Usability Questionnaire
QUIS: Questionnaire for User Interaction Satisfaction
SDG: Sustainable Development Goal
SEIPS: Systems Engineering Initiative for Patient Safety
SU-ISERC: Strathmore University Institutional Scientific Ethics Review Committee
SUS: System Usability Scale
UX: user experience
