# Supplementary material for: Usability and Workflow Integration of a Machine Learning–Derived Neonatal Risk Predictor in Kenyan Neonatal Units: Multisite User-Centered Pilot Evaluation
Source: JMIR Hum Factors. 2026 Sep 9;13:e94828. doi: 10.2196/94828 (PMC13601881; doi:10.2196/94828)
Supplement: Supplementary file 1 [file humanfactors_v13i1e94828_app1.pdf]

S1 Table: Good Reporting of A Mixed Methods Study (GRAMMS) checklist

Item	Reported
Describe the justification for using a mixed-methods approach to the research question	A mixed-methods approach was used because evaluating the implementation of a neonatal risk predictor required both quantitative usability outcomes and qualitative contextual explanations. Triangulation provided a comprehensive understanding of usability, workflow integration, and implementation.
Describe the design in terms of the purpose, priority, and sequence of methods	Embedded post-implementation cross-sectional mixed-methods evaluation. After a four-month implementation period, quantitative surveys (n=10) were completed first, followed by qualitative KIIs (n=3), with integration during interpretation.
Describe each method in terms of sampling, data collection, and analysis	Quantitative: census of available frontline nurses/midwives using SUS, PSSUQ, QUIS, and PSNUQ; descriptive analysis. Qualitative: purposive sampling of neonatal unit leaders; semi-structured interviews; thematic analysis with iterative coding and consensus.
Describe where integration has occurred, how it has occurred, and who has participated in it	Integration occurred during interpretation through triangulation of survey findings and KII themes to explain usability, workflow integration, implementation barriers, and clinical utility.
Describe any limitation of one method associated with the presence of the other method	The quantitative sample was small (n=10), qualitative interviews involved only three key informants, and thematic saturation was not formally assessed. Mixed-methods integration strengthened interpretation despite these limitations.
Describe any insights gained from mixing or integrating methods	Triangulation showed that positive usability ratings were influenced by contextual factors, including staffing shortages, documentation burden, governance support, and workflow conditions, leading to practical implementation recommendations.

Reference: O'Cathain A, Murphy E, Nicholl J. The quality of mixed methods studies in health services research. J Health Serv Res Policy. 2008;13(2):92-98.
