# Supplementary material for: Usability and Workflow Integration of a Machine Learning–Derived Neonatal Risk Predictor in Kenyan Neonatal Units: Multisite User-Centered Pilot Evaluation
Source: JMIR Hum Factors. 2026 Sep 9;13:e94828. doi: 10.2196/94828 (PMC13601881; doi:10.2196/94828)
Supplement: Supplementary file 2 [file humanfactors_v13i1e94828_app2.pdf]

Annex 2: Key Informant Interview (KII) Questionnaire

Thank you for taking time out of your busy schedule to participate in this study titled: ***Kenyan Neonatal Mortality Risk Predictor: A User-Centred Design Evaluation***. The aim of the study is to explore the Perceived purpose, current situation, potential benefits, implementation approach, and potential barriers to implementation of the proposed Neonatal Mortality Risk (NMR) Screening Tool in health facilities providing maternal services in low-and middle-income countries (LMICs). This will help to determine the feasibility of the tool in reducing neonatal mortality in the Kenyan context. By doing so, the study aligns with Kenya's health priorities, and thus contributing to global efforts to reduce neonatal mortality towards achieving target 3.2 of

Part A: Understanding of the NMR Screening Tool and its Purpose (Questions 1-4)

1. What is your understanding of the purpose and goals of the proposed neonatal risk-screening tool?
2. What do you perceive to be the role/use of this tool within the broader context of neonatal care?
3. What are your expectations regarding contribution of the tool in neonatal care?
4. Who would be the users of the tool?

Part B: Understanding the Current Situation (Questions 1-6)

1. What is the size of your neonatal care unit in your facility?
2. What is the total number of neonatal care staff in your facility, and what are their professions?
3. How many deliveries are recorded per day in your facility on average?
4. What method(s) do you use to identify high-risk neonates to prioritize their care and treatment?
5. How many referrals do you make per week?
6. How many neonatal deaths are recorded per week in your facility?

Part C: Potential Benefits of NMR Screening Tool (Questions 1-3)

1. Do you feel the proposed tool should be able to facilitate prioritized and timely triage-decisions for high-risk neonates in your facility?
2. Do you feel the tool should improve productivity of neonatal staff in your facility?
3. Do you feel the tool should improve neonatal health outcomes?

Part D: Preferred Implementation Approach (Questions 1-3)

1. Do you feel the language of the tool should be contextualized?
2. What neonatal risk factors do you feel should be used in the NMR screening tool?
3. Do you feel the NMR screening tool should be paper-based or digital? Give reasons for your answer.

Part E: Potential barriers to Implementing NMR Screening Tool (Questions 1-5)

1. What do you feel would be potential barriers to the successful implementation and sustained use of the proposed tool?
2. Are there any resource constraints that hinder the tool's effectiveness?
3. Are there any cultural or social factors will impact the tool's acceptance and use in your facility?
4. Are there any policy related challenges that will hinder the implementation of the tool?
5. How can these barriers be addressed to ensure the tool's long-term sustainability

Thank you for your time and feedback.

Annex 2: Risk Factors for Intrapartum Stillbirth and Neonatal Mortality Survey

This survey incorporates neonatal risk factors identified as significant predictors at two time points: Delivery/Day 1 and Post-delivery/Day 2.

Instructions: Please complete the following questions based on the mother and neonate's information. Accurate information is crucial for accurate risk assessment.

Section 1: Maternal Information

1. **Maternal Age:** What is the mother's age in years? _______ years
2. **Parity:** How many times has the mother previously given birth to a baby (live or stillborn) after 20 weeks of gestation? _______
3. **Maternal Education:** Did the mother receive any formal schooling?
   - Yes
   - No *(This specifically addresses the predictor "Maternal education: none")*
4. **Hypertension/Severe Pre-eclampsia/Eclampsia:** During this pregnancy, did the mother have high blood pressure (recorded as \geq 140/90 mmHg), severe pre-eclampsia, or eclampsia (seizures)?
   - Yes
   - No
   - Unknown
5. **Suspected Maternal Sepsis:** During labor or delivery, was there suspected maternal sepsis (indicated by fever plus pelvic pain and abnormal vaginal discharge, potentially foul-smelling)?
   - Yes
   - No
   - Unknown
6. **Maternal Antibiotics:** Did the mother receive antibiotics during labor or immediately after delivery?
   - Yes
   - No
   - Unknown

Section 2: Pregnancy Information

1. **Gestational Age at Enrollment:** At how many weeks of gestation did the mother first attend antenatal care for this pregnancy? _______ weeks
2. **Multiple Birth:** Was this a pregnancy with more than one baby (e.g., twins, triplets)?
   - Yes
   - No
3. **Antenatal Corticosteroids:** Did the mother receive antenatal corticosteroid injections before delivery?
   - Yes
   - No
   - Unknown
4. **Antepartum Hemorrhage:** Did the mother experience any vaginal bleeding after 24 weeks of pregnancy but before labor started?
   - Yes
   - No
   - Unknown

Section 3: Delivery Information

1. **Gestational Age at Delivery:** At how many completed weeks of gestation did the delivery occur? _______ weeks
2. **Obstructed Labor:** Was the labor diagnosed as obstructed (labor not progressing despite adequate contractions)?
   - Yes
   - No
   - Unknown
3. **Delivery Location:** Was the baby delivered in a hospital?
   - Yes
   - No *(e.g., clinic, home)*
4. **Delivery Attendant:** Was the delivery primarily managed/conducted by a physician?
   - Yes
   - No *(e.g., midwife, traditional birth attendant)*
   - Unknown
5. **Cluster Perinatal Mortality:** *(Note: This information typically comes from facility/regional data, not individual assessment. It refers to the background risk level in the specific service delivery area.)*

Section 4: Neonatal Information (Delivery / Day 1)

1. **Birth Weight:** What was the neonate's weight at birth? _______ grams
2. **Neonatal Sex:** What is the sex of the neonate?
   - Male
   - Female
3. **Birth Order:** (Only for multiple births) Was this the first, second, or later-born baby from this pregnancy? _______ (e.g., 1, 2, 3...)
4. **Bag and Mask Resuscitation:** Did the neonate require breathing support using a bag and mask immediately after birth?
   - Yes
   - No

Section 5: Post-Delivery Information (Day 2)

1. **Neonatal Hospitalization:** Was the neonate admitted to a hospital ward or neonatal unit within the first 2 days after birth?
   - Yes
   - No
2. **Neonatal Antibiotics:** Did the neonate receive antibiotic medication within the first 2 days after birth?
   - Yes
   - No

Important Considerations:

- **Using the Survey:** This survey collects data points identified as predictors. To *predict* risk, the collected data would need to be input into the specific predictive model developed in the original study (mentioned as a gradient boosted ensemble model in the image notes). The survey itself gathers the inputs; it doesn't perform the risk calculation.
- **Data Source:** Some information (like maternal history or delivery specifics) might be best obtained from the mother, while other details (birth weight, resuscitation, neonatal treatments) would come from clinical records or healthcare providers.
- **Cluster Perinatal Mortality:** This specific predictor reflects the baseline risk in the geographic area or facility and requires external data, not typically gathered through an

individual survey.

- **Definitions:** Ensure clear, standardized definitions (like those for hypertension and sepsis provided in the image footnote) are used when administering the survey.

Annex 3: General Survey Questionnaire

Thank you for taking time out of your busy schedule to participate in this study titled: ***Kenyan Neonatal Mortality Risk Predictor: A User-Centred Design Evaluation***. The aim of the study is to evaluate the usability, user experience (UX) and utility of the Neonatal Mortality Risk (NMR) Screening Tool in health facilities providing maternal services in low-and middle-income countries (LMICs). This will help to determine the feasibility of the tool i.e. ensuring effective and efficient identification of high-risk infants to prioritize their care & treatment to effectively control neonatal mortality risk factors in the Kenyan context. By doing so, the study aligns with Kenya's health priorities, and thus contributing to global efforts to reduce neonatal mortality towards achieving target 3.2 of Sustainable Development Goal 3 (SDG 3).

This survey uses validated scales in parts A, B, C, & D below to gather user opinions on the **usability**, **UX**, and **Utility** of the NMR Screening Tool provided and tested earlier on.

PART A: Participant Demographics

Please indicate (tick/choose the appropriate option):

1. What is your gender?
   Male ☐ Female ☐ Other ……………………………………………………
2. What is your age?
   18-30 ☐ 31-40 ☐ 41-50 ☐ 51-60 ☐ > 60 years ☐
3. What is your highest attained academic qualification?
   Certificate/Diploma ☐ Bachelor's degree ☐ Master's degree ☐ PhD degree ☐
4. What is your profession?
   Midwife ☐ Nurse ☐ Clinical Officer ☐ General Practitioner ☐ Obstetrician ☐

   Other……………………………………………….
5. What is your current job position/level/rank in your organization? ………………………………
6. What is your work experience in neonatal care?
   1-5 ☐ 6-10 ☐ 11-15 ☐ 16-20 ☐ > 21 years ☐
7. What is your main role in your organization/department/? ……………………………………………

PART B: Usability Scale Questionnaire (USQ) for Tool Evaluation

The RAEng Neonatal Risk Predictor Research Project

Purpose: This questionnaire evaluates your experience in using the NMR Screening Tool based on the structure of the Questionnaire for USQ.

Instructions: Please rate the following statements based on your experience using the NMR Screening Tool.

Scale:
1 = Strongly Disagree
2 = Disagree
3 = Neutral
4 = Agree
5 = Strongly Agree

Statements:

1. I would like to use this tool frequently [1 2 3 4 5]

2. I found the tool unnecessarily complex [1 2 3 4 5]

3. I thought the tool was easy to use [1 2 3 4 5]

4. I think that I would need assistance to use this tool [1 2 3 4 5]

5. I found the various NMR factors in this tool well-presented [1 2 3 4 5]

6. I thought there was too much inconsistency in this tool [1 2 3 4 5]

7. I would imagine that most people would learn to use this tool very quickly [1 2 3 4 5]

8. I found the tool very cumbersome/awkward to use [1 2 3 4 5]

9. I felt very confident using the tool [1 2 3 4 5]

10. I needed to learn a lot of things before I could get going with this tool [1 2 3 4 5]

11. I can recommend this tool to someone else [1 2 3 4 5]

12. I won't recommend this tool to anyone [1 2 3 4 5]

Comments:..
....
....
....

Thank you for your feedback.

PART C: Questionnaire for UX and Interaction Satisfaction (QUIS) for Tool Evaluation

Purpose: This questionnaire evaluates your UX and Interaction Satisfaction with the NMR Screening Tool based on the structure of the Questionnaire for QUIS

Instructions: For each item below, please circle the number that best represents your opinion. The numbers range from 1 (strongly associated with the adjective on the left) to 9 (strongly associated with the adjective on the right). Use 5 for neutral.
If a question does not apply to this tool or your experience, please mark N/A.

Section 1: Overall Reaction to the NMR Screening Tool (Questions 1-6)

Rate your overall reaction to using this tool in MNR assessment:

1	Terrible	[1 2 3 4 5 6 7 8 9]	Wonderful	N/A
2	Difficult	[1 2 3 4 5 6 7 8 9]	Esy	N/A
3	Frustrating	[1 2 3 4 5 6 7 8 9]	Satisfying	N/A
4	Inadequate Power	[1 2 3 4 5 6 7 8 9]	Adequate Power	N/A
5	Dull	[1 2 3 4 5 6 7 8 9]	Stimulating	N/A
6	Rigid	[1 2 3 4 5 6 7 8 9]	Flexible	N/A

Comments:..
....
....
....

Section 2: Text Layout (Questions 7-10)
Regarding the content:

7	Characters hard to read	[1 2 3 4 5 6 7 8 9]	Character easy to read	N/A
8	Cluttered Layout	[1 2 3 4 5 6 7 8 9]	Clean Layout	N/A
9	Frustrating	[1 2 3 4 5 6 7 8 9]	Satisfying	N/A
10	Confusing information organization	[1 2 3 4 5 6 7 8 9]	Clear information organization	N/A

Comments:..
....
....
....

Section 3: Terminology and NMR Screening Tool Information (Questions 11-15)

Regarding the language, terminology, and terms used in the NMR Screening Tool:

11	The language is not understandable	[1 2 3 4 5 6 7 8 9]	The language is understandable	N/A
12	Terminology is inconsistent	[1 2 3 4 5 6 7 8 9]	Terminology is consistent	N/A
13	Specific terms confusing	[1 2 3 4 5 6 7 8 9]	MCH-specific terms clear	N/A
14	Information inconsistent	[1 2 3 4 5 6 7 8 9]	Messages consistent	N/A
15	Information unhelpful	[1 2 3 4 5 6 7 8 9]	Messages helpful	N/A

Comments:...
....
....
....

Section 4: Learning the NMR Screening Tool (Questions 16-20)

Regarding how easy it is to learn and remember how to use the tool for daily job tasks:

16	Learning the tool is difficult	[1 2 3 4 5 6 7 8 9]	Learning the tool is easy	N/A
17	Remembering terminology is difficult	[1 2 3 4 5 6 7 8 9]	Remembering terminology easy	N/A
18	Understanding specific terms is difficult	[1 2 3 4 5 6 7 8 9]	Understanding specific terms is easy	N/A
19	Tasks can be performed straightforwardly: never	[1 2 3 4 5 6 7 8 9]	Tasks can be performed straightforwardly: Always	N/A
20	Information unhelpful	[1 2 3 4 5 6 7 8 9]	Messages helpful	N/A

Comments:...
....
....
....

Section 5: NMR Screening Tool's Capabilities (Question 20)

Regarding the performance and capabilities of the tool:

21	The tool meets workflow needs poorly	[1 2 3 4 5 6 7 8 9]	The tool meets neonatal care workflow needs well	N/A

Comments:...
....
....
....

Thank you for your detailed feedback!

PART D: Post-Study NMR Tool Utility Questionnaire (PSNUQ) – Tool Evaluation

Purpose: This questionnaire assesses your satisfaction with the utility of NMR Screening Tool after completing specific job tasks-based on the structure of the Questionnaire for PSNUQ (Version 3). This evaluation questionnaire assesses how well the tool meets your user needs and its overall utility. It includes questions about ease of use, clarity of instructions, and relevance of content, and overall effectiveness to identify areas of improvement to make the tool user friendly and efficient.

Instructions: Please rate your agreement with each statement below based on your experience.
Use the 7-point scale where:
1 = Strongly Agree
2 = Agree
3 = Slightly Agree
4 = Neutral
5 = Slightly Disagree
6 = Disagree
7 = Strongly Disagree

Lower scores indicate greater utility and vice versa. Mark N/A if the statement is not applicable.

Section 1: The NMR Screening Tool's Usefulness (Questions 1-6)

1. Overall, I am satisfied with how easy it is to use this tool [1 2 3 4 5 6 7] [NA]

2. It was simple to use this tool [1 2 3 4 5 6 7] [NA]

3. I can effectively complete my work using this tool [1 2 3 4 5 6 7] [NA]

4. I feel comfortable using this tool [1 2 3 4 5 6 7] [NA]

5. It was easy to learn to use this tool [1 2 3 4 5 6 7] [NA]

6. I believe I could become productive quickly using this tool [1 2 3 4 5 6 7] [NA]

Comments:..
....
....
....

Section 2: Content Quality of the NMR Screening Tool (Questions 7-14)

7. The content in this tool is relevant to my daily work [1 2 3 4 5 6 7] [NA]

8. The language used in this tool is appropriate for the intended audience [1 2 3 4 5 6 7] [NA]

9. The information in this tool is up-to-date and accurate [1 2 3 4 5 6 7]

10. The information in the tool is complete [1 2 3 4 5 6 7]

11. The information in this tool was clear and concise [1 2 3 4 5 6 7] [NA]

12. It was easy to find the information I needed [1 2 3 4 5 6 7] [NA]

13. The information in this tool was effective in helping me complete my job tasks [1 2 3 4 5 6 7] [NA]

14. The organization of information in this tool was clear [1 2 3 4 5 6 7] [NA]

Comments:..
....

Section 3: Interface Quality of the NMR Screening Tool (Questions 15-17)

15. The interface of this tool was pleasant [1 2 3 4 5 6 7] [NA]

16. I liked using the interface of this tool [1 2 3 4 5 6 7] [NA]

17. This tool has all the NMR factors and capabilities I expected it to have [1 2 3 4 5 6 7] [NA]

Comments:..
....

Section 4: Impact and Effectiveness of the NMR Screening Tool (Questions 18-21)

18. Has the tool helped to identify newborns at risk more effectively than previous methods?

Yes ☐ **No** ☐ If yes, how different was the tool compared with previous methods?

19. Have you noticed any changes in the referral patterns for neonates after the introduction of the tool? If yes, what are these changes in referral patterns?

Yes ☐ **No** ☐

20. What is the nature of the impact of the tool on the timeliness of interventions (care and treatment) for at-risk neonates?

High impact ☐ Medium impact ☐ Low impact ☐ Not sure ☐

21. Have there been any positive or negative consequences associated with using the tool?
Yes ☐ **No** ☐ If yes, what are the specific positive consequences?

Comments:...
....

Section 5: Overall Satisfaction (Question 21)

21. Overall, I am satisfied with this tool [1 2 3 4 5 6 7] [NA]

Comments:...
....

PART E: Potential Barriers to Implementation and Sustainability of the NMR Tool (Questions 1-3)

1. What are the potential barriers to the successful implementation and sustained use of the tool?

Difficulty to learn ☐ Difficulty terminology ☐ Poor user interface ☐

Paper-based nature ☐ User/staff resistance ☐ Organizational culture ☐

Resource constraints ☐

Other ....

2. Are there any cultural or social factors that impact on the tool's acceptance and use?

Yes ☐ **No** ☐ If yes, what are the specific factors identified?

3. How can these barriers be addressed to ensure the tool's long-term sustainability?

Comments:...
....

PART F: Potential Improvement of the NMR Tool (Questions 1-7)

1. What changes would you recommend to improve the tool's effectiveness?

Language ☐ Terminology ☐ NMR tool's variables ☐ Create a digital version ☐

Other...

2. Are there any new features, elements or risk factors that you feel would enhance the tool's utility? **Yes** ☐ **No** ☐ If yes, which specific ones?

3. Do you feel the tool needs to be adapted to better suit the specific needs of different LMIC healthcare settings and populations? **Yes** ☐ **No** ☐ If yes, how should this be done?

4. Do you feel further research or evaluation is needed to assess the tool's long-term impact?

Yes ☐ **No** ☐ If, yes, which aspects of the tool should be evaluated further?

5. What is your understanding of the goals and purpose of the NMR screening tool?

Goals: Early intervention and treatment ☐ Minimizing the impact of risk factors ☐

Purpose: Identification of high-risk neonates ☐ Timely triage decisions ☐

Other……

6. Can you provide an example of how the tool can be better used to identify a newborn at risk?

………

Comments:……
………

Thank you for your time and feedback.
