# Supplementary material for: Usability and Workflow Integration of a Machine Learning–Derived Neonatal Risk Predictor in Kenyan Neonatal Units: Multisite User-Centered Pilot Evaluation
Source: JMIR Hum Factors. 2026 Sep 9;13:e94828. doi: 10.2196/94828 (PMC13601881; doi:10.2196/94828)
Supplement: Supplementary file 3 [file humanfactors_v13i1e94828_app3.pdf]

Verbatim Quotes by Theme

1. Risk Identification & Screening

Kibra: "Basically what I've come to understand about the purpose of this tool is to really aid in terms of early detection of any baby or any neonate who is at risk. And also be able to use the predictors to guide us in terms of initiation of early care, early referral and provision of timely management, which can really aid in minimizing the risk."

Nazaret: "This tool will help us identify the high-risk babies and high-risk pregnancies. And it will help us in management, like future management, because once you identify a high-risk baby or pregnancy, you are already in a position to plan the management earlier."

Matuu: "We use the APGAR score after delivery. We also use gestational age, especially for preterm babies who are prone to infection, so that we can identify those who need close monitoring or referral."

2. Digital Systems & Data

Kibra: "I prefer the paperless system because when we say paper-based, sometimes there is a problem with storage and also it provides extra work in terms of writing. Sometimes you find somebody does not even have a pen, and that information might be left out, which is very crucial."

Nazaret: "Me, I would prefer digital because it will be easy to enter and easy to analyze. And you are very sure that by the end of the day you will not lose any of your documents, because with the computer-based one there is data backup."

Matuu: "We are currently using Afya One, Excel, and some paperwork. So it is a combination of digital systems and manual records, depending on what data is being captured."

3. Unit Capacity & Workload

Kibra: "We are understaffed most of the time. Sometimes somebody might see this tool as a bigger workload, because already we have other forms of documentation, and the staff are few compared to the number of patients."

Nazaret: "If we are under-staffed, I do what is essential and leave what is not. By the end of the day, you prioritize patient care and skip some of the documentation because you cannot do everything."

Matuu: "Ideally one nurse should care for two patients, but currently one nurse may handle up to ten patients. This makes it very difficult to give the required level of attention to every neonate."

4. Infrastructure & Resources

Kibra: "Electricity and network issues are a challenge. Our Wi-Fi is not reliable, and we depend solely on Kenya Power. When there is a power outage, it affects our service provision because we do not have a functional backup generator."

Nazaret: "The number of computers may not be enough if we want to implement a digital system. It requires capital, and installing and maintaining these systems needs money."

Matuu: "Staff shortages are the main challenge, and they also affect how well we can use any new system that is introduced."

5. Policy & Governance

Kibra: "The county policies are generally supportive. If the county approves and says go ahead, then implementation becomes easier for us at the facility level."

Nazaret: "We don’t have very specific policies guiding the use of computerized systems in neonatal care. Sometimes the facility may be willing, but if the county is not ready, then it becomes a challenge."

Matuu: "The tool will be used by trained healthcare workers, and county policies are generally supportive, so there are no major policy barriers that we anticipate."

6. Training & Attitude

Kibra: "Some of the people are really reluctant to change. They might see this as something that is bringing more work to them, especially when staffing levels are already low."

Nazaret: "Training is really required because this is a new tool. If the training is done adequately and staff are well informed about its importance, it will really help improve their attitude towards using it."

Matuu: "The language of the tool is simple and easy to understand. As long as staff are trained on how to use it, acceptance will not be a problem."
